# Gut microbiota and metabolites exhibit different profiles after very-low-caloric restriction in patients with type 2 diabetes

**DOI:** 10.3389/fendo.2023.1289571

**Published:** 2024-01-10

**Authors:** Tong Gong, Hongjie Di, Yongxin Hu, Shuhang Xu, Jie Chen, Guofang Chen, Xiao Wei, Chao Liu

**Affiliations:** ^1^ Department of Endocrinology, Jiangsu Province Second Hospital of Chinese Medicine, Second Affiliated Hospital of Nanjing University of Chinese Medicine, Nanjing, China; ^2^ Department of Endocrinology, Affiliated Hospital of Integrated Traditional Chinese and Western Medicine, Nanjing University of Chinese Medicine, Nanjing, China; ^3^ Jiangsu Province Academy of Traditional Chinese Medicine, Nanjing, China; ^4^ Department of Nutrition, Affiliated Hospital of Integrated Traditional Chinese and Western Medicine, Nanjing University of Chinese Medicine, Nanjing, China

**Keywords:** type 2 diabetes, very-low-calorie restriction, insulin resistance, gut microbiota, metabonomics

## Abstract

**Background and aims:**

To investigate the effect of short-term very-low-calorie restriction (VLCR) on metabolism in patients with type 2 diabetes (T2D), and elucidate the molecular mechanism through analyses on gut microbiota and small-molecule metabolites.

**Methods:**

Fourteen T2D patients were hospitalized to receive VLCR (300-600 kcal/d) for 9 days. BMI, BP, and HR were taken before and after VLCR. Levels of blood lipids, fasting insulin, FBG, and 2h PBG were assessed. The microbial diversity in feces was detected by 16S rDNA high-throughput sequencing technology, and small-molecule metabolites in plasma and feces by untargeted metabolomics technology.

**Results:**

After VLCR, BW, BMI, WC, BP, and levels of FBG and 2h PBG, insulin, HOMA-IR, and triglyceride decreased significantly in T2D patients (*P*<0.05). There was no significant change in the α-diversity of fecal microbiota, but the abundance of *Bacteroidetes* increased significantly, and the *Firmicutes/Bacteroidetes* ratio decreased significantly from 11.79 to 4.20. *Parabacteroides distasonis* showed an abundance having increased most prominently after VLCR treatment. Plasma level of amino acid metabolite L-arginine increased significantly. Plasma levels of three lipid metabolites, PC (14:0/20:4 [8Z, 11Z, 14Z, 17Z]), LysoPC (16:1 [9Z]) and LysoPC (18:1 [11Z]), were significantly reduced. Fecal levels of lipid metabolite LysoPC (18:1 [11Z]) and bile acid metabolite glycholic acid were significantly decreased.

**Conclusion:**

In T2DM patients, VLCR can considerably reduce body weight and improve glucose and lipid metabolism without causing severe side effects. LysoPC (18:1 [11Z]) and *Parabacteroides distasonis* showed the most obvious difference after VLCR, which could be the indicators for VLCR in T2D.

## Introduction

Calorie restriction (CR) is being used to treat an array of diseases, such as aging-associated diseases (1). There is a growing body of evidence pointing to the benefits of CR for glucolipid metabolism. Glucose and lipid homeostasis can be improved by a slight weight loss, which, according to current dietary recommendations, is most often achieved through energy reduction (2). Given the interplay between human obesity and T2DM, CR has been trialed to alleviate T2DM. Low-calorie diets (825-853 kcal/d) for three to five months can dramatically reduce body weight and HbA1c level in T2DM patients (3). Also, after a two-year follow-up period, one-third of T2DM patients reported relief, with their HbA1c levels kept below 6.5% without the use of hypoglycemic medications (4). Our previous studies have yielded similar results (5, 6).

However, the mechanism underpinning this efficacy is still unclear. Taylor believes that this is related to the dissolution of the twin cycle hypothesis and the remission of fatty liver and pancreas (7). So far, many animal experiments have shown that CR may improve glucolipid metabolism and insulin resistance by reshaping gut microbiota (8), the composition of which is closely related with diet (9, 10). When its composition is disrupted, unusual gut microbial metabolites will be produced, such as short-chain fatty acids and bile acids (11). These metabolites can serve as molecules signaling to body metabolism (12).

Against this background, our study aimed to identify the key microbes and metabolites associated with very-low-calorie restriction (VLCR), and the molecular mechanism of VLCR in improving glucolipid metabolism in T2DM. In the meantime, non-targeted metabolomics technology was utilized to detect small molecule metabolites in plasma and fecal samples, and 16S rDNA high-throughput sequencing technology was employed to assess gut microbial diversity in fecal samples before and after VLCR.

## Materials and methods

### Participants

Fourteen individuals with T2DM were recruited by advertisement. T2DM was diagnosed according to the criteria of WHO (1999) (13). Included were those with an age 18-65 years, a BMI 24-40 kg/m^2^, fasting blood glucose ranged from 7.0 to 16.7 mmol/L and a duration less than 10 years. Exclusion criteria were HbA_1c_ >12.0%; hemoglobin ≤ 100 g/L, neutrophil <1.5×10^9^/L; myocardial enzyme profile (Creatine kinase CK and creatine kinase isoenzyme CK-MB) ≥3 × Upper limit of normal (ULN); glutamic-pyruvic transaminase ≥2.5×ULN and/or glutamic-oxalacetic transaminase ≥ 2.5×ULN and/or total bilirubin ≥1.5×ULN (except for metabolic related fatty liver disease); renal function injury defined as eGFR <60 mL/min/1.73 m^2^; fasting triglyceride ≥5.64 mmol/L (500 mg/ dL); a history of gastrointestinal diseases and gastrointestinal surgery; intake of antibiotics in previous 3 months and probiotic foods in previous 1 week. All participants stopped anti-diabetic therapy two days before VLCR, but still maintained regular lipid-lowering therapy. Antihypertensive medications were decreased as necessary throughout the study. The study protocol was approved by the Chinese Clinical Trial Registry (ChiCTR1800018199), and all participants gave informed written consent.

### Experimental protocol

Participants were given a normal diet for 1-2 days after admission. VLCR started after routine physical examination and laboratory examination during this period. According to 2016 Dietary Guidelines for Chinese Residents, VLCR was designed to contain 55% carbohydrate, 20% protein and 25% fat, and prescribed over three periods, including buffer period, restriction period and recovery period. Buffer period and recovery period lasted 2 days (300 kcal/d). The restriction period lasted 5 days (600 kcal/d). Participants were guided to drink at least 2 L of water per day and to maintain their physical habits. During VLCR, blood glucose, blood pressure and heart rate were monitored daily. Any case of hypoglycemia (blood glucose ≤ 3.9 mmol/L) was treated with 20 g of glucose or equivalent food supplement (calculated according to daily calorie supply), and measured for blood glucose again 15 minutes later. The morning urine ketone body was measured during the restriction period to monitor the subject's compliance. Blood sample was taken on fasting state in the morning of the first day of the VLCR and on the first day after the VLCR. Fecal sample was taken 1-2 days before the VLCR and 1-2 days after the VLCR, and must be the first stool after the VLCR.

### Anthropometric data

Patients’ height and weight were measured by physicians and body mass index (BMI) was calculated (weight in kilograms divided by the square of the height in meters). Height and waist circumference were measured at an accuracy level of 0.01 cm by a stadiometer (Suhong Medical Device Ltd, Jiangsu, China), and weight at an accuracy level of 0.01 kg by an SUHONG RGZ-160 scale (Suhong Medical Device Ltd, Jiangsu, China). According to the guideline for Chinese adults, 18.5 kg/m^2^ ≤ BMI < 24 kg/m^2^ was considered as normal weight, BMI ≥ 24 kg/m^2^ as overweight, and BMI ≥ 28 kg/m^2^ as obesity.

### Glucose and lipids

After fasting for 10 hours overnight, the venous blood was drawn by a nurse in the morning. Roche cobas 8000 (Roche Diagnostics Ltd, Shanghai, China) was used to measure the levels of fasting blood glucose (FBG), total cholesterol (TC), triglyceride (TG), high-density lipoprotein cholesterol (HDL-C), low-density lipoprotein cholesterol (LDL-C), calcium (Ca), phosphorus (P), albumin (ALB), ALT, AST, ALP, γ-GT, Cr, and UA. The level of hemoglobin Alc (HbAlc) was measured with the high pressure liquid chromatography method. Roche cobas 602 (Roche Diagnostics Ltd, Shanghai, China) was used to analyze the levels of fasting insulin (FINS). Insulin resistance (IR) and β-cell function were indirectly ascertained by the Homeostasis Model Assessment (HOMA) as follows: HOMA-IR=FBG (mmol/L)×FINS (mIU/L) /22.5; HOMA-β=20×FBG (mmol/L) / (FINS [mIU/L] -3.5) (%).

### Fecal microbiota

Total DNA was extracted from the feces according to the instructions of the E.Z.N.A.® soil kit (Omega Bio-Tek, USA). DNA concentration and purity were detected by NanoDrop2000 (Thermo Fisher Scientific, USA). Sequencing was performed using Miseq PE300 platform of Illumina (Shanghai Magi Biomedical Technology Co., LTD.), and the raw data were uploaded to NCBI database. The original sequencing sequence was quality-controlled by Trimmomatic software and spliced by FLASH software. A cluster of operational taxonomic units (OTUs) operated on sequences based on 97% similarity using UPARSE software. The RDP classifier was used to annotate each sequence, and the comparison threshold was set to 70% for Silva database.

### Metabolites in the plasma and feces

The untargeted metabolomic analysis was used to detect metabolites in the plasma and feces. The instrument platform of LC-MS analysis was UHPLC-Q Exactive system of Thermo Fei. The original data were imported into the metabolomic processing software Progenesis QI (Waters Corporation, Milford, USA) for baseline filtering, peak recognition, integration, retention time correction and peak alignment. Finally, a data matrix of retention time, mass charge ratio and peak area was obtained, and then the data were preprocessed. Only the variables with more than 50% non-zero value in all samples were retained, and the missing values were filled with 1/2 of the minimum value in the original matrix. Then the total peak was normalized, and the variables with relative standard deviation ≥30% of the quality control samples were deleted. Then, log10 transformation was performed to obtain the data matrix for subsequent analysis. The mass spectrometry information was matched with that in the metabolic database. The main databases included public databases and self-built databases.

### Statistical analysis

All data were analyzed using the SPSS SamplePower software, version 24.0 (IBM Corporation, Chicago, IL). Baseline characteristics were presented as means ± SD or as median (interquartile range [IQR]). The data before and after VLCR were compared with student paired and two-sample t test and Wilcoxon rank sum test. Statistical significance was set at a *P*-value < 0.05.

## Results

### Effects of VLCR on body weight and plasma metabolites

A total of 16 patients were recruited from January 2019 to December 2020 to participate in the study, among which 1 patient withdrew from the study on the 4th day due to unbearable hunger, and 1 patient was also excluded because he did not get feces after the experiment. On the whole, the relevant indicators of 14 patients (8 males and 6 females) were enrolled. Among them, 9 patients had new onset T2DM, and the remaining 5 patients had a history of 5.34±3.42 years. Mean (SD) values of body weight, waist circumference and HbA1c were 96.46±20.51 kg, 107.79±11.21 cm and 8.08±3.41%, respectively.

After 9 days of VLCR, the mean body weight fell from 96.46±20.51 kg at baseline to 91.50±18.99 kg (*P*<0.001). Waist circumference fell from 107.79±11.21 cm to 102.82±10.21 cm (*P*<0.001). Fasting plasma glucose level fell from 8.49±2.69 to 5.42±1.28 mmol/L (*P*=0.001). Fasting insulin level decreased form 16.76 (9.52, 23.72) to 7.89 (4.92, 13.36) mIU/L (*P*=0.035). Insulin resistance was reduced significantly when HOMA-IR fell from 6.20 (3.21, 10.31) to 1.92 (1.21, 4.11) (*P*=0.002). Lipid metabolism was also partially improved when triglyceride level decreased from 2.46±1.06 to 1.54±0.66 mmol/L (*P* <0.001). VLCR brought no significant changes in the levels of TC, HDL-C, LDL-C, AST, ALT, γ-GT, Cr, eGFR, UA, ALB, Ca and P (*P*>0.05) (**Tables 1**, **2**).

**Table 1:** Changes of anthropometric measurements and glycolipid parameters in T2DM patients after VLCR (n=14).

Parameters	Before CR	After CR	*P* value
BW (kg)	96.46±20.51	91.50±18.99	<0.001
BMI (kg/m^2^)	33.71±4.69	31.87±4.43	<0.001
WC (cm)	107.79±11.21	102.82±10.21	<0.001
SBP (mmHg)	132.00±13.81	124.64±9.28	0.005
DBP (mmHg)	83.93±12.48	74.79±8.88	0.001
HR (bpm)	83.43±11.80	77.79±5.06	0.042
FBG (mmol/L)	8.49±2.69	5.42±1.28	0.001
2hPBG (mmol/L)	12.94±3.63	8.36±2.14	<0.001
FINS (µIU/ml)	16.76 (9.52, 23.72)	7.89 (4.92, 13.36)	0.035
HOMA-IR	6.20 (3.21, 10.31)	1.92 (1.21, 4.11)	0.002
TG (mmol/L)	2.46±1.06	1.54±0.66	<0.001
TC (mmol/L)	4.73±1.22	4.33±1.15	0.121
HDL-C (mmol/L)	0.95±0.29	0.87±0.14	0.187
LDL-C (mmol/L)	3.13±1.03	3.28±1.11	0.426

Data are mean (SD) or median (IQR).

**Table 2:** Changes of liver and kidney function and electrolyte indexs in T2DM patients after VLCR (n=14).

Parameters	Before CR	After CR	*P* value
AST (U/L)	26.86±14.85	32.50±17.88	0.113
ALT (U/L)	41.36±25.62	43.86±32.27	0.718
ALP (U/L)	80.57±20.31	74.14±14.95	0.045
γ-GT (U/L)	42.50 (16.65, 60.75)	36.45 (21.50, 50.95)	0.054
Cr (µmol/L)	69.07±18.17	72.43±14.90	0.087
BUN (mmol/L)	5.43±1.68	4.09±1.65	0.005
eGFR (ml/(min·1.73 m^2^)	109.64±29.13	107.62±27.00	0.476
UA (µmol/L)	410.71±158.98	461.57±148.43	0.148
ALB (g/L)	44.61±3.63	43.38±3.08	0.329
Ca (mmol/L)	2.27±0.09	2.27±0.11	0.801
P (mmol/L)	1.20±0.21	1.28±0.19	0.105

Data are mean (SD) or median (IQR).

### Effects of VLCR on fecal microbial diversity

**Figure 1:**
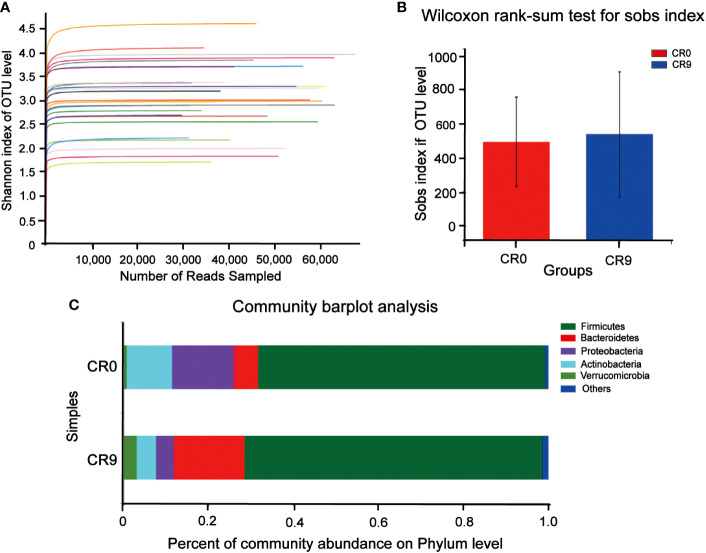
**(A)** Shannon curve. **(B)** Gut microbiota α diversity before and after VLCR. **(C)** Major phylum before and after VLCR.

We compared the composition of gut microbiota using 16S rRNA analysis. Shannon indices showed that the sequencing results could reflect the microbial diversity in the samples (**Figure 1A**). Wilcoxon rank-sum test was performed for sobs index. As shown in **Figure 1B**, after VLCR, the α-diversity in gut microbiota was slightly increased, but without statistical significance (*P*> 0.05). Partial least squares-discriminant analysis (PLS-DA) on the phylum level showed differences in gut microbiota (**Figure 1C**). The number of species on the phylum level went from 37 to 42 after 9 days of VLCR treatment. The five new species were *Armatimonadetes*, *Fibrobacteres*, *Kiritimatiellaeota*, *Margulisbacteria* and *FCPU426*. Among them, the proportions of *Armenterobacteria* and *FCPU426* were 61.11% and 5.56%, respectively; the proportion of the abundance of *Fibrillary bacteria*, *Kiritimatiellaeota* and *Marguliciaceae* was 11.1%.

### Effects of VLCR on fecal microbial composition

**Figure 2:**
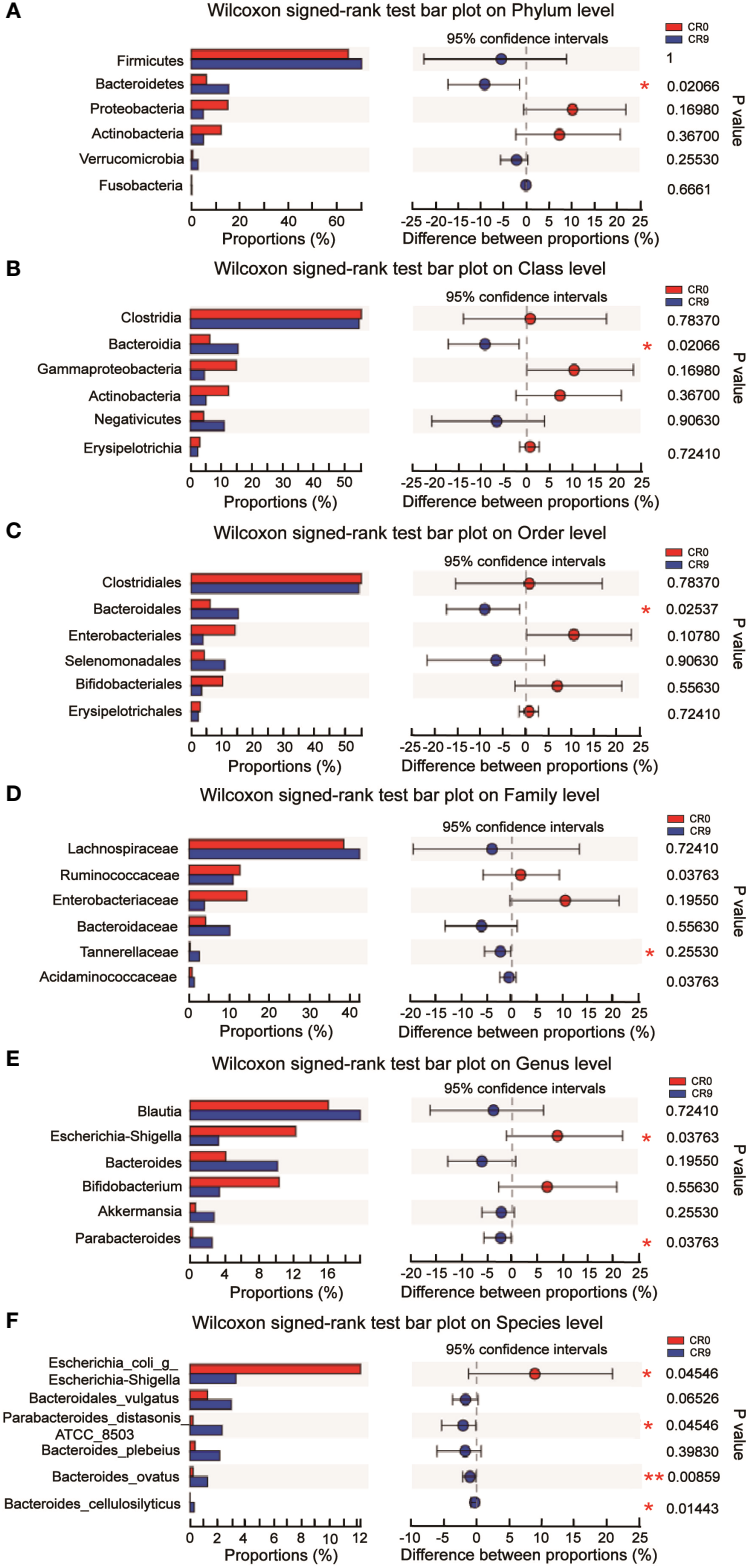
**(A)** Difference at the level of phylum before and after VLCR. **(B)** Difference at the level of class before and after VLCR. **(C)** Difference at the level of order before and after VLCR. **(D)** Difference at the level of family before and after VLCR. **(E)** Difference at the level of genus before and after VLCR. **(F)** Difference at the level of species before and after VLCR. *, P<0.05; **, P<0.01; ***, P<0.001.

The community barplot analysis on the phylum level indicated that the abundance of *Bacteroidetes* increased significantly from 5.73% to 16.70 % (*P*< 0.05). The abundance of *Firmicutes* increased from 67.57% to 70.13%, with no statistical significance. However, the abundance of *Firmicutes/Bacteroidetes* decreased from 11.79 to 4.20 significantly (*P*<0.05). The abundances of *Actinobacteria*, *Proteobacteria* and *Verrucomicrobia* also changed, but without statistical significance. Wilcoxon signed-rank test barplots showed that *Bacteroidia*, *Bacteroidales* and *Tannerellaceae* families increased significantly in abundance. On the genus level, the abundance of *Escherichia-Shigella* decreased significantly, and hat of *Parabacteroides* increased significantly. At the species level, *e. coli/Shigella abundance* decreased significantly, and those of *Parabacteroides distasonis*, *Bacteroides_ovatus* and *Bacteroides_cellulosilyticus* increased significantly (*P*<0.05) (**Figure 2**).

### Key species and their links with clinical indicators

**Figure 3:**
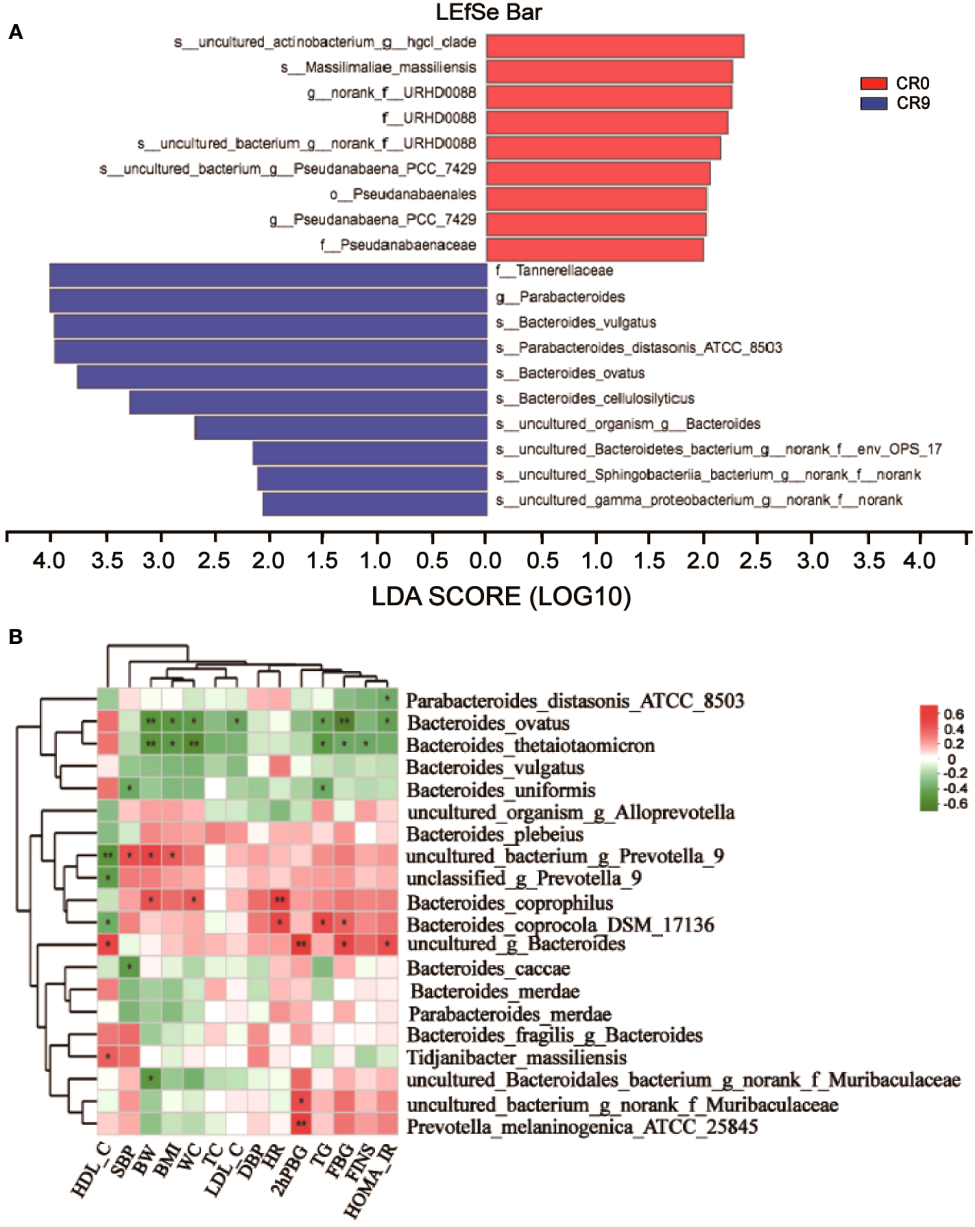
**(A)** LEfSe analysis on phylum, class, order, family, genus and species. **(B)** Correlation analysis of gut microbiota and metabolic indexes. *, P<0.05; **, P<0.01.

We performed LEfSe and Linear discriminant analysis (LDA) to estimate the key species. *Tannerellaceae* and *Parabacteroides* had the highest LDA scores after VLCR, both of which were 4.014, followed by *Bacteroides_vulgatus* (3.971) and *Parabacteroides distasonis* (3.965) (**Figure 3A**). As shown in **Figure 3B**, the abundance of *Parabacteroides_distasonis_ATCC_850* was negatively correlated with HOMA-IR (*P*<0.05). The abundance of *Bacteroides_ovatus* was negatively correlated with HOMA-IR, FBG, TG, LDL-C, WC, BMI and BW (*P*<0.05). However, the abundance of *Bacteroides_vulgatus* had no significant correlation with clinical metabolic indexes (*P*>0.05).

### Effects of VLCR on plasma metabolites

**Figure 4:**
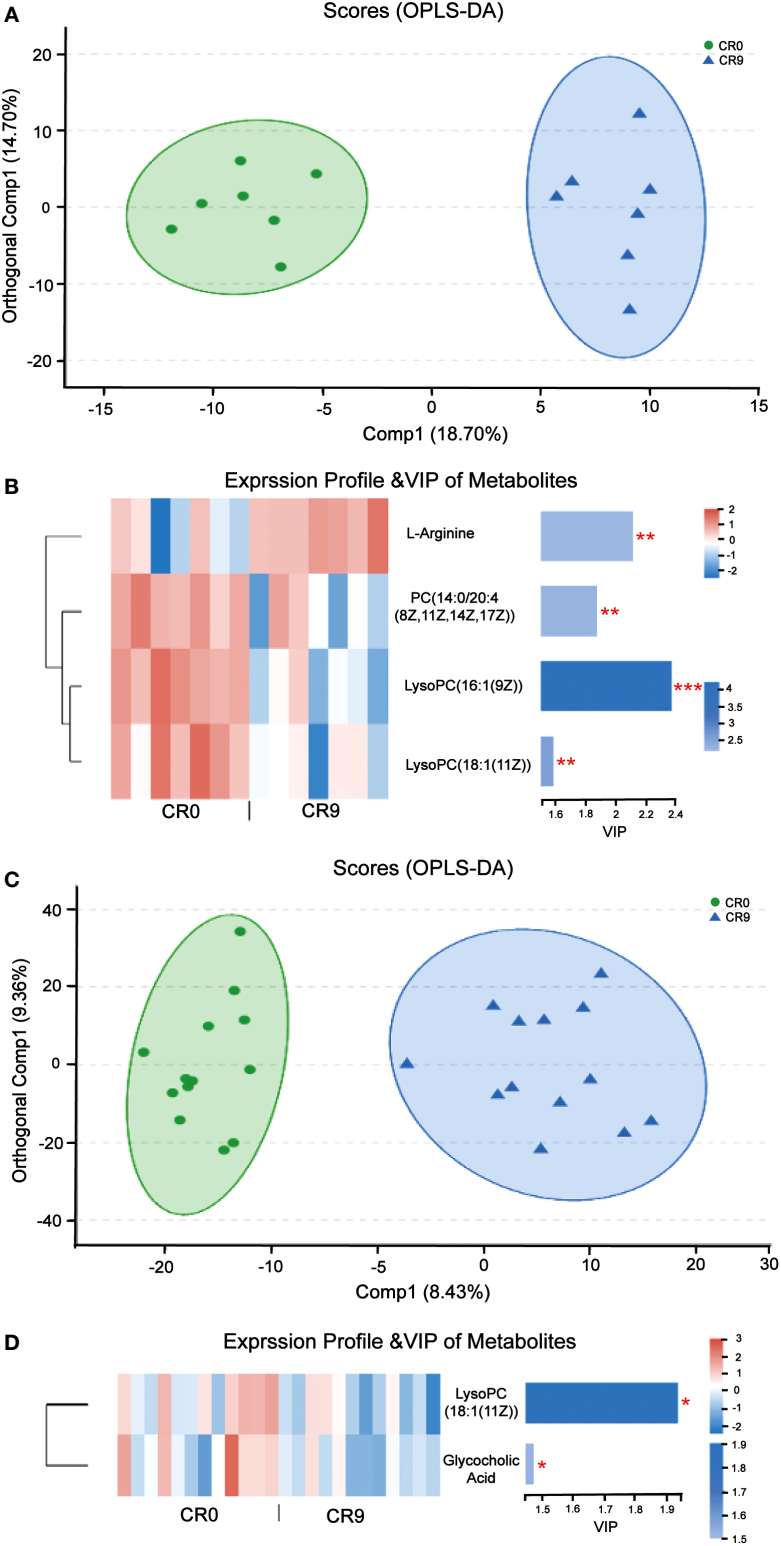
**(A)** OPLS-DA of plasma metabolites before and after VLCR. **(B)** The significant changes of plasma metabolites before and after 9-day VLCD. **(C)** OPLS-DA of fecal metabolites before and after caloric restriction. **(D)** The significant changes of fecal metabolites before and after 9-day VLCD. *, P<0.05; **, P<0.01; ***, P<0.001.

Partial least squares discriminant analysis (PLS-DA) and orthogonal partial least squares discrimination analysis (OPLS-DA) indicated that the metabolites in plasma changed significantly after VLCR (**Figure 4A**). According to VIP values obtained in OPLS-DA, differential metabolites between groups were screened out (VIP>1, *P*< 0.05), and finally four metabolites with biological significance were obtained. Among them, three belongs to the type of lipid metabolites: LysoPC (16:1 [9Z]), PC (14:0/20:4 [8Z, 11Z, 14Z, 17Z]) and LysoPC (18:1 [11Z]) in VIP order. The plasma levels of these three metabolites decreased significantly after VLCR. The other differential metabolite, L-Arginine, as an amino acid, increased significantly after VLCR (**Figure 4B**).

### Effects of VLCR on fecal metabolites

PLS-DA and OPLS-DA indicated that fecal metabolites changed significantly after VLCR (**Figure 4C**). According to VIP values obtained in OPLS-DA, the differential metabolites between groups were screened out (VIP> 1, *P*< 0.05), and finally two small molecule metabolites with biological significance were obtained. The level of LysoPC (18:1 (11Z)), a lipid metabolite, dropped significantly after VLCR. The level of glycholic acid, a bile acid-like metabolite, also decreased significantly after VLCR (**Figure 4D**).

### Discussion

This study demonstrated a significant improvement in glycolipid metabolism in T2DM patients after a 9-day VLCR. The microbial abundances on all levels showed remarkable changes. The lipid and amino acid metabolites in the plasma and feces underwent considerable alterations.

The diversity of fecal microbiota somewhat increased following VLCR (P > 0.05). Five more kinds of bacteria, namely *Armatimonadetes*, *Fibrobacteres*, *Kiritimatiellaeota*, *Margulisbacteria*, and *FCPU426* appeared at the phylum level. The five can enhance cellulose hydrolysis (14–17). An essential dietary fiber, cellulose serves as the foundation of plant cell walls. The amount of dietary fiber rises in the VLCR diet, and the levels of microorganisms that encourage cellulose hydrolysis increase in tandem. Therefore, VLCR can rapidly change the structure of gut microbiota in T2DM patients.

The abundance of *Bacteroidetes* considerably increased after VLCR (*P*<0.05). *Bacteroidetes* have been revealed directly involved in metabolic diseases (18). *Bacteroidetes* are less prevalent in obese mice, the weight of which can be reduced by supplement with multiform *Bacteroidetes* (18). Following VLCR, the abundance of *Bacteroidetes* and *Bacteroidales* dramatically increased in the present study. LEfSe and Linear Discriminant Analysis showed that the LDA scores of *Tannerellaceae*, *Parabacteroides*, and *Parabacteroides distasonis* were 4.014, 4.014, and 3.965, respectively, indicating the most notable elevations in their abundances. The relationships among *Tannerellaceae*, *Parabacteroides* and *Parabacteroides distasonis* are superior and subordinate, and all belong to the phylum *Bacteroides.* The main bacteria at the species level are *Tannerellaceae* and *Parabacteroides*. The abundance of *Parabacteroides distasonis*, one of the primary symbiotic bacteria in the intestinal tract, is adversely linked with the severity of inflammatory bowel disease, non-alcoholic fatty liver disease, and obesity (19). *Parabacteroides distasonis* has a substantially low level in high-fat-fed mice (20). Furthermore, body weight, glucose level, and hepatic steatosis show beneficial changes in obese mice treated with active *Parabacteroides distasonis* (21). Consistently, the present study proved that the efficacy of VLCR on T2MD can be partly attributed to *Parabacteroides distasonis* in the enhancement of glucolipid metabolism.

The plasma level of L-arginine increased significantly after VLCR. In diabetic patients, a significant decrease in plasma L-arginine concentration is observed (22). Basic studies suggest that L-arginine can activate AMP-activated protein kina (AMPK), then stimulate fatty acid oxidation and glucose uptake in skeletal muscles, thereby increasing insulin secretion (23).

On the contrary, plasma levels of lipid metabolites PC (14:0/20:4 [8Z, 11Z, 14Z, 17Z]), LysoPC (16:1 [9Z]) and LysoPC (18:1 [11Z]) were significantly reduced after VLCR. Among them, PC (14:0/20:4 [8Z, 11Z, 14Z, 17Z]) belongs to phosphatidylcholines (PC) which is closely associated with T2DM. High intake of dietary PC content may increase the risk of T2DM by 17% (24). After gastric bypass, serum PC level in T2DM patients decreased significantly (25). LysoPC (16:1 [9Z]) and LysoPC (18:1 [11Z]) are lysophosphatidylcholine (LPC). LPC is an important group of lipid molecules in mammalian tissues, and LPC with different structures carry intertissue fatty acids, phosphatidylglycerol and choline (26). Researchers have observed a higher level of LPC in obese people (27). Plasma LysoPC (18:1) level in diabetic patients is also significantly higher than that in prediabetic patients (28). Taken together, upregulation of PC (14:0/20:4 [8Z, 11Z, 14Z, 17Z]), LysoPC (16:1 [9Z]) and LysoPC 18:1 [11Z]) may promote the occurrence and development of obesity and diabetes. Supportively, the present study found that VLCR can downregulate these metabolites to alleviate the symptoms of T2DM.

Here, VLCR significantly suppressed the fecal levels of LysoPC (18:1 [11Z]) and glycocholic acid (GCA), a conjugated primary bile acid produced by combining cholic acid and glycine (29). The serum level of GCA is higher in patients with liver disease than in healthy individuals (30). The linkage between GCA and obesity has also been verified by recent research. The fecal levels of GCA at 3 and 12 months following gastric bypass surgery are much lower than that prior to surgery in obese T2DM women (31). The role of GCA in metabolic illnesses has been rarely reported. This study indicates that it may be targeted to intervene metabolic diseases relevant to T2DM and obesity.

Remarkably, the LysoPC (18:1) levels in both the plasma and feces demonstrated similar decreasing trends following VLCR. Studies have revealed that LPC directly promotes inflammation by inducing the production and release of interleukin-1 (IL-1) (32). In the meantime, the nuclear factor-activated B cells are triggered to create pro-inflammatory effects, which enhances the nuclear factor-K-gene binding K (NF-KB) signaling pathway (33). Besides, procytokines can be transformed into active forms of IL-1, IL-18, and IL-33 by casparase-1, when stimulated by LPC-dependent activation of nicotinamide adenine dinucleotide phosphate oxidase and generation of reactive oxygen species (34). During VLCR, the LysoPC (18:1) levels in the plasma and feces decreased, implying the mitigation of inflammation in the body, which further alleviates T2MD. It is also believed that reduced inflammation after VLCR is directly linked to a higher abundance of *Parabacteroides distasonis*. Mice fed with *Parabacteroides distasonis* produce fewer pro-inflammatory cytokines, such as tumor necrosis factor-alpha (TNF-alpha), which represses intestinal inflammation and restores gut microbiota (35). We therefore speculate that the efficacy of VLCR on T2MD may involve a crosstalk between *Parabacteroides distasonis*, LysoPC (18:1) and inflammatory factors.

In conclusion, VLCR can significantly reduce body weight, blood glucose and triglycerides, and weaken insulin resistance in patients with T2DM. VLCR may function mainly through increasing the abundance of *Parabacteroides distasonis*, a dominant bacterium in the intestinal tract, and decreasing the LysoPC (18:1) levels in the plasma and feces.

### Limitations of study

There are also some deficiencies in this study. First, the study did not include healthy controls, so we should further analyze the differences in gut microbiota and metabolites between T2DM patients and healthy controls, as well as the effect of VLCR on these differences. Second, the sample size was small and should be expanded in the future. Finally, no basic experiments were performed to prove the relationship between *Parabacteroides distasonis* and LysoPC (18:1). We will clarify this relationship, and provide more theoretical evidence for VLCR in the treatment of T2DM.

## Data availability statement

The data presented in the study are deposited in the National Center for Biotechnology Information repository, accession number PRJNA1014817.

## Ethics statement

The studies involving humans were approved by the Ethics Committee of Affiliated Hospital of Integrated Traditional Chinese and Western Medicine, Nanjing University of Chinese Medicine. The studies were conducted in accordance with the local legislation and institutional requirements. The participants provided their written informed consent to participate in this study.

## Author contributions

TG: Data curation, Formal analysis, Investigation, Methodology, Visualization, Writing – original draft. HD: Investigation, Methodology, Writing – review & editing. YH: Investigation, Methodology, Writing – review & editing, Resources. SX: Investigation, Writing – review & editing, Data curation, Formal analysis. JC: Investigation, Writing – review & editing, Methodology, Resources. GC: Supervision, Writing – review & editing, Conceptualization, Funding acquisition, Resources. XW: Project administration, Supervision, Writing – review & editing, Validation, Visualization. CL: Conceptualization, Project administration, Supervision, Writing – review & editing.
